# Multi-region sampling with paired sample sequencing analyses reveals sub-groups of patients with novel patient-specific dysregulation in Hepatocellular Carcinoma

**DOI:** 10.1186/s12885-022-10444-3

**Published:** 2023-02-03

**Authors:** Ah-Jung Jeon, Yue-Yang Teo, Karthik Sekar, Shay Lee Chong, Lingyan Wu, Sin-Chi Chew, Jianbin Chen, Raden Indah Kendarsari, Hannah Lai, Wen Huan Ling, Neslihan Arife Kaya, Jia Qi Lim, Adaikalavan Ramasamy, Gokce Oguz, Alexander Yaw-Fui Chung, Chung Yip Chan, Peng-Chung Cheow, Juinn Huar Kam, Krishnakumar Madhavan, Alfred Kow, Iyer Shridhar Ganpathi, Tony Kiat Hon Lim, Wei-Qiang Leow, Shihleone Loong, Tracy Jiezhen Loh, Wei Keat Wan, Gwyneth Shook Ting Soon, Yin Huei Pang, Boon Koon Yoong, Diana Bee-Lan Ong, Jasmine Lim, Vanessa H. de Villa, Rouchelle D.dela Cruz, Rawisak Chanwat, Jidapa Thammasiri, Glenn K. Bonney, Brian K. P. Goh, Greg Tucker-Kellogg, Roger Sik Yin Foo, Pierce K. H. Chow

**Affiliations:** 1 Department of Hepatopancreatobiliary and Transplant Surgery, Division of Surgery and Surgical Oncology, Singapore General Hospital and National Cancer Centre Singapore, Singapore, Singapore; 2 Program in Clinical and Translational Liver Cancer Research, Division of Medical Science, National Cancer Center Singapore, Singapore, Singapore; 3 Genome Institute of Singapore, Agency for Science, Technology and Research (A*STAR), Singapore, Singapore; 4 Academic Clinical Programme for Surgery, Duke-NUS Medical School, Singapore, Singapore; 5 Division of Hepatobiliary & Pancreatic Surgery, Department of Surgery, University Surgical Cluster, National University Health System, Singapore, Singapore; 6 Department of Anatomical Pathology, Singapore General Hospital, Singapore, 169608 Singapore; 7 Department of Pathology, National University Hospital, Singapore, 119074 Singapore; 8 Department of Surgery, Faculty of Medicine, University of Malaya, Kuala Lumpur, Malaysia; 9 Department of Surgery and Center for Liver Health and Transplantation, The Medical City, Pasig City, Metro Manila Philippines; 10 Department of Laboratory Medicine and Pathology, The Medical City, Pasig City, Metro Manila Philippines; 11 Hepato-Pancreato-Biliary Surgery Unit, Department of Surgery, National Cancer Institute, Bangkok, Thailand; 12 Division of Pathology, National Cancer Institute, Bangkok, Thailand; 13 Department of Biological Sciences, National University of Singapore, Singapore, Singapore; 14 Cardiovascular Disease Translational Research Programme, Yong Loo Lin School of Medicine, National University of Singapore, Singapore, Singapore; 15 Cardiovascular Research Institute, Yong Loo Lin School of Medicine, National University of Singapore, Singapore, Singapore

**Keywords:** Multi-region sampling, Patient subgroups, Personalized medicine

## Abstract

**Background:**

Conventional differential expression (DE) testing compares the grouped mean value of tumour samples to the grouped mean value of the normal samples, and may miss out dysregulated genes in small subgroup of patients. This is especially so for highly heterogeneous cancer like Hepatocellular Carcinoma (HCC).

**Methods:**

Using multi-region sampled RNA-seq data of 90 patients, we performed patient-specific differential expression testing, together with the patients’ matched adjacent normal samples.

**Results:**

Comparing the results from conventional DE analysis and patient-specific DE analyses, we show that the conventional DE analysis omits some genes due to high inter-individual variability present in both tumour and normal tissues. Dysregulated genes shared in small subgroup of patients were useful in stratifying patients, and presented differential prognosis. We also showed that the target genes of some of the current targeted agents used in HCC exhibited highly individualistic dysregulation pattern, which may explain the poor response rate.

**Discussion/conclusion:**

Our results highlight the importance of identifying patient-specific DE genes, with its potential to provide clinically valuable insights into patient subgroups for applications in precision medicine.

**Supplementary Information:**

The online version contains supplementary material available at 10.1186/s12885-022-10444-3.

## Background

Hepatocellular carcinoma (HCC) is the most dominant type of primary liver cancer and is one of the most important causes of cancer-related deaths globally [1]. HCC is an intricately complicated disease with aetiologies that include chronic viral hepatitis B/C, chronic cirrhosis from any cause, non-alcoholic fatty liver diseases, genetic dispositions, toxin exposures, and autoimmune liver diseases. The combination of these elements results in variations of the disease that have distinct molecular profiles across patients [2]. The use of systemic monotherapies in such a heterogenous cancer has shown limited efficacy; first line drugs such as Sorafenib and Lenvatinib have best overall response rates (BORR) of less than 20% [3], while the combination therapy atezolizumab and bevacizumab has marginally better BORR of 30% for patients with advanced HCC [4]. One of the challenges in developing therapies for HCC patients has lain in the diversity of the tumour.

Tumour diversity extends from the heterogeneity observed between patients (inter-individual heterogeneity) to the heterogeneity within tumours of individual patients (intra-tumoural heterogeneity). The challenge posed by cancer heterogeneity has prompted the adoption of a multi-region sampling approach in several cancer studies [5, 6], including the prospective PLANet study which focused on elucidating intra-tumoural heterogeneity in HCC (NCT03267641). Genomics and immunomics analyses of multi-region tumour sampling have yielded new insights into both tumour heterogeneity and tumour evolution [7].

Much of cancer subtyping has currently been done based on phenotypic differences that arise from the inter-individual heterogeneity. Subtyping often involves identifying key genetic signatures or protein markers that can group patients based on the molecular profiles of their tumour tissues. Different molecular markers have been used to define tumour subtypes, such as the presence of surface markers or proteins [8], different immune cell population [9], or the expression level of selected marker genes [10]. The conventional approach in cancer subtyping and in broad cancer studies using RNA-seq typically involves identifying differentially expressed (DE) genes in the tumour tissues compared to the normal tissues. Many well-established statistical methods are available [11, 12] and their general approach is to fit a linear model for each gene and perform a statistical testing to select genes with distinct separation between the tumour and normal samples. These approaches successfully identify genes differentially expressed across the cohort, but they may be less effective in detecting dysregulation in individual patients’ tumour. While outlier detection methods are sometimes used to identify genes with unusual expression in a subset of patients [13], individual patients are rarely the focus of analysis. This is often a necessity, as most studies are limited to a single tumour sample and a single normal sample per patient, or only include a subset of the patients’ normal tissues under the assumption that the adjacent normal tissues would be similar between patients.

In this study, our aim was to perform differential expression analysis per patient, to first identify dysregulated genes in each patient then identifying dysregulated genes in a subgroup of patients. We used the multi-region sampling data from the PLANet study (NCT03267641) to perform patient-level transcriptomic analysis by treating the multi-region samples as biological replicates. The PLANet is a prospective cohort of patients with surgically resected HCC from which multiple samples were obtained from individual tumours as previously described [7, 14]. In addition to the previously published dataset which included 44 patients [14], we also report additional dataset from 46 patients that were collected and processed using the same pipeline. We hypothesized that per-patient analyses applied to all patients would identify more dysregulated genes in total than all-patients analysis, and that those genes unique to the per-patient analyses would highlight the inherent differences among patients. We compared conventional DE analysis, patient-specific analysis and downstream aggregation of patient-specific differential expression, revealing subgroup-specific DE genes that elude conventional analysis. The subgroup-specific genes were then used to stratify patients into subgroups with differential prognosis. Patient-specific transcriptional dysregulation identified from multi-region tumour sequencing has the potential to provide clinically valuable insights into patient subgroups for applications in precision medicine.

## Materials and methods

### Patient recruitment and sample preparation

Our dataset includes 90 anonymized patients with 344 tumour samples and 90 normal samples obtained from the ongoing PLANet cohort study (NCT03267641) (Additional file 1). The multi-region tumour samples were obtained from surgically resected liver tumour by harvesting a single slice through the capsule along one axis of the tumor and the normal sample were obtained from the adjacent normal liver tissue (≥ 2 cm away) from the tumor, as described in [7]. This prospective cohort is deeply phenotyped and does not harbour any treatment prior to resection. Material from each patient contains at least 2 tumour samples and 1 normal sample. All patients had HCC confirmed by histology and full clinical trajectory for recurrence analysis.

### RNA-seq data

**Fig. 1:**
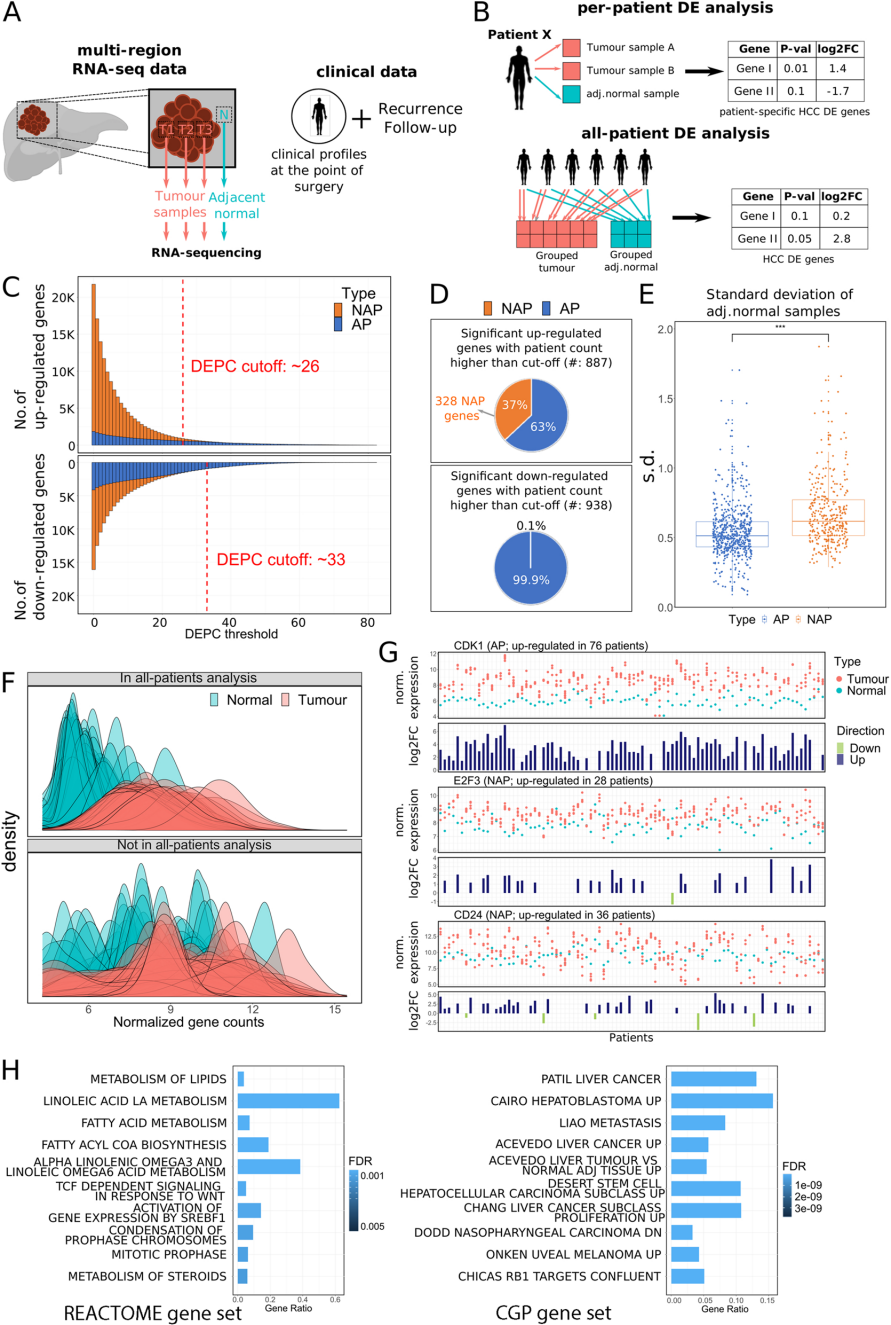
Per-patient analysis identified patient specific dysregulated genes. **A**: Data acquisition overview for multi-region RNA-seq data and clinical data of patients. **B**: Comparison between all-patients and per-patient DE analyses. **C**: Number of up-/down-regulated genes above a given DEPC threshold against different DEPC thresholds. DEPC cutoff for up-/down-regulated genes was determined based on two standard deviations. **D**: Visualization of the proportion of AP vs NAP genes in up-/down-regulated genes above the respective DEPC thresholds. **E**: Standard deviations of normal gene expression of AP genes against NAP genes. **F**: Overlayed density plots of tumour and normal gene expression of the top 50 AP/NAP genes with highest DEPC counts. **G**: Normalized gene expression and their respective log2FoldChange values from per-patient analysis of example genes (CDK1, CD24 & WNT5A). Only CDK1 was also found to be up-regulated in all-patients analysis. **H**: GSEA results for the 328 up-regulated NAP genes against the REACTOME (left) and CGP gene sets (right) from MSigDB. Top 10 enriched gene sets were shown

**Fig. 2:**
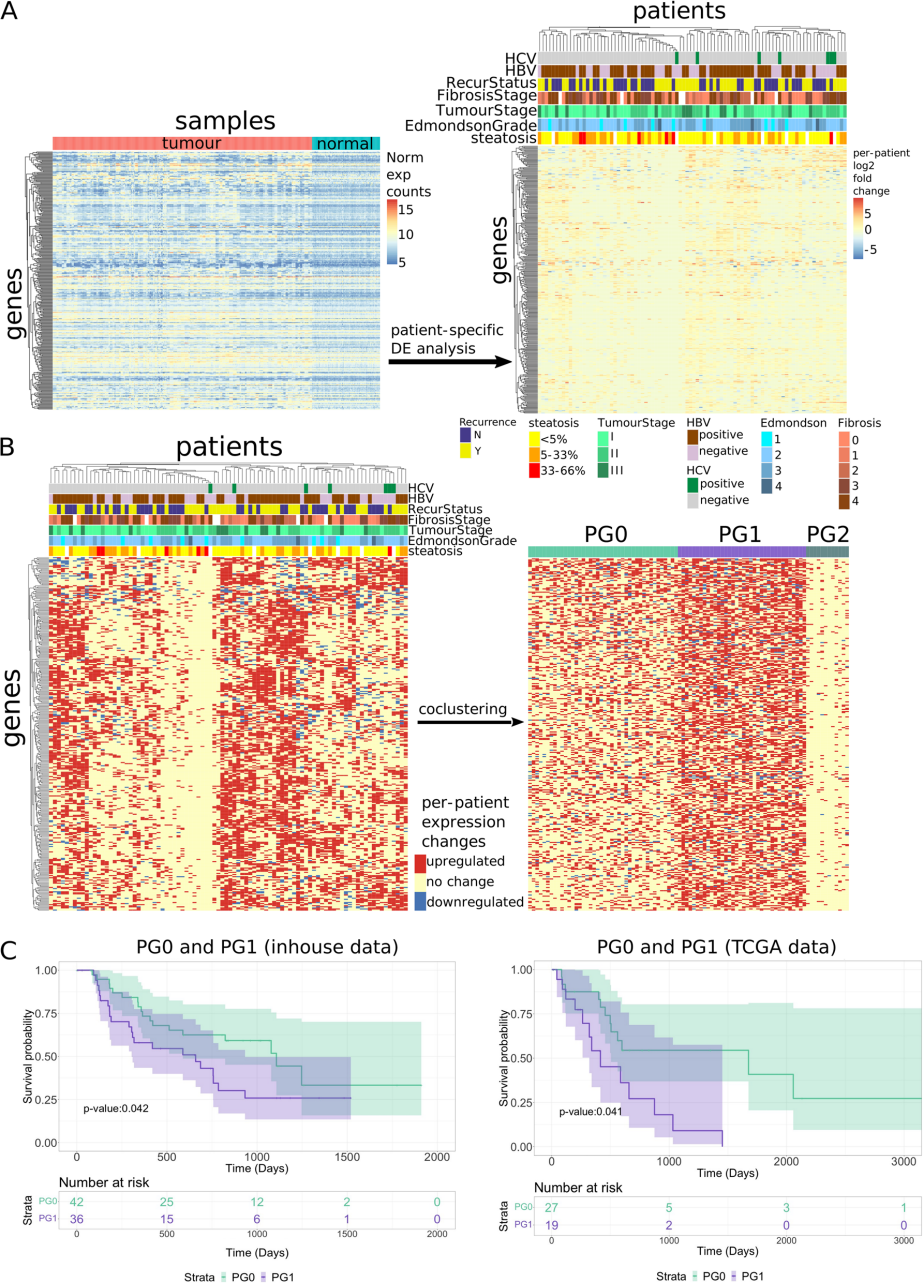
Patient stratification using patient-specific expression changes. **A**: Left—Normalized gene expression of the 328 NAP genes for all samples. The samples are first grouped by the sample type and ordered within each sample type. Right—Log2FoldChange matrix of the 328 NAP genes based on per-patient DE analysis. **B**: Left—Categorical matrix of the NAP genes based on per-patient DE analysis. Right – Co-clustered categorical expression changes heatmap, generated after applying co-clustering to the categorical matrix on the left. All rows in all of the above mentioned four heatmaps, and the columns in the three patient expression changes heatmaps (A right, B left, B right) are based on hierarchical clustering of the categorical expression changes heatmap (B, left). **C**: Left—Disease-free (Recurrence-free) plot of patients in PG0 and PG1 using the clinical data of the patients. Right—Progression-free Kaplan–Meier plot of patients classified as PG0 and PG1 from the TCGA-LIHC cohort, using the model built on our data. More details shown in Fig. S3A

All samples were prepared following the same protocols as described in [7]. RNA-seq data was mapped to hg38 genome build [15] and GENCODE annotation [16] using STAR pipeline and the raw read counts quantified using RSEM were normalized using DESeq2 along with log2 transformation as described in [7]. RNA-seq data for Fig. 1G and RNA-seq data for gene count heatmap visualization in Fig. 2A and Fig. 2B were vst-normalized. RNA-seq data used for multi-layer perceptron (MLP) classification was Trimmed Mean of *M*-values (TMM) counts-per-million (CPM)-log normalization using the edgeR R package.

### Differential gene expression analysis

Differential gene expression analysis was performed using the DESeq2 R package [12], and the genes were filtered for at least 1 count in 30% of the samples. For all-patients analysis, the model matrix design included both the sample type (tumour vs normal) as well as the patient ID. For per-patient analysis, the model matrix design consisted of only the type of sample. Log-fold change shrinkage for both all-patients and per-patient analysis was formulated with the apeglm package. The model matrix design for the differential expression analysis between patients in PG0 and PG1 consists of the patient groups, interaction variable between patient group and nested patient, and interaction variable between patient group and sample type. Log-fold change shrinkage was obtained using the ashr package. Default parameters were used for the DESeq2. Genes were considered as differentially expressed if the adjusted *p*-value is less than 0.05 and the absolute value of log2FoldChange is more than 1.

### Gene set enrichment analysis with MSigDB gene sets

Gene set enrichment analysis was performed using the hypergeometric test with R base package. Gene sets were downloaded from MSigDB (REACTOME and CPG) v7.4. Gene sets were considered enriched if the false-discovery-rate value was less than 0.05.

### Co-clustering & survival analysis

Co-clustering was performed using the blockcluster R package [17]. Survival analysis was performed using the survival and survminer R packages [18, 19]. All survival analysis *p*-values were obtained with the modified Peto-Peto test. Disease-free survival analysis was done using recurrence-free survival days in our dataset, while progression-free interval (PFI) days were used for TCGA dataset, as recommended by TCGA guidelines.

### Statistical testing

Association between PGs and clinical variables were performed using fisher’s exact or chi-squared independence tests for categorical variables and two-sided Wilcoxon tests for continuous variables. All other statistical tests between two groups were one-sided Wilcoxon tests unless stated otherwise.

### TCGA-LIHC RNA-seq data

The Cancer Genome Atlas-Liver Hepatocellular Carcinoma (TCGA-LIHC) data was downloaded using the TCGAbiolinks R package [20]. The RNA-seq data obtained was the HTSeq counts. Clinical variables were downloaded from the TCGA browser.

### Data visualization

Data visualization was done using the following R packages: ggplot2 [21], gridExtra [22], cowplot [23], pheatmap [24], ggsignif [25], and ggpubr [26].

### R & Python packages and libraries versions

All the analyses in R were performed using R version 4.0.3. The R packages used were DESeq2 version 1.32.0, edgeR version 3.32.0, blockcluster version 4.5.0, survival version 3.2.7, survminer version 0.4.8, TCGAbiolinks version 2.18.0, ggplot2 version 3.3.2, gridExtra version 2.3, cowplot version 1.1.0, pheatmap version 1.012, ggsignif version 0.6.0 and ggpubr version 0.4.0. All Python analysis is performed using python version 3.8.2. The Python libraries used are torch version 1.5.0, sklearn version 0.22.1. numpy version 1.18.1, pandas version 1.0.3, and csv version 1.0.

### Assessment of fibrosis, steatosis and microvascular invasion

The fibrosis in the non-tumoural liver was staged according to the meta-analysis of histological data in viral hepatitis (METAVIR) staging system from stages F0 to F4, based on histological evaluation of both haematoxylin & eosin (H&E) stained-slides and Masson Trichrome stained-slides (if available). Steatosis was graded based on the percentage of parenchymal involvement by macrovesicular steatosis from grades 0 to 3, based on histological evaluation of H&E stained slide. Microvascular invasion is defined as the presence of tumour cells within and adherent to the vessel wall, either covered by endothelium or in a context of thrombus or fibrin, based on histological evaluation of H&E stained slide.

### Data preparation for MLP classification

Normalized RNA-seq data are paired such that each tumour sample is paired with the respective normal sample. Subsequently, taking the difference between the tumour and normal gene expression, we obtained the Tumour-Normal-Difference (TND) data. While the original number of NAP genes we selected was 328, we only selected 327 genes as our training features. This is to ensure that the training features are coherent between our dataset and the TCGA-LIHC dataset. For the training data, each training instance of a patient is labelled with the PG that the patient belongs to. For example, if Patient X has 3 tumour samples and 1 normal sample, we can obtain 3 TND training instances and the 3 training instances are labelled with the PG that Patient X belongs to.

### MLP classification model

The MLP model is built using PyTorch. The model consists of 4 hidden linear layers, of which, the first 3 layers utilize Kaiming initialization and relu activation, and the last layer uses Xavier initialization and softmax activation. The model also uses stochastic gradient descent (SGD) optimization with a learning rate of 0.01 and momentum of 0.9, and cross-entropy loss for training. The training is performed across 500 epochs with a batch size of 32. The training–testing ratio is 7:3.

### Classification and prediction results

We performed 1000 trials of the MLP classification task. For each trial, the dataset derived from our RNA-seq counts is randomly split into the training and testing set. Using the testing set, we can calculate the classification accuracy of the MLP model for each trial. Subsequently, we then used the model to predict the label (PG) for each instance of TND data derived from the TCGA-LIHC data. Across the 1000 trials, we observed that the predicted labels of the same instance may vary. To test if the predictions of a single instance are random, we performed the chi-square goodness of fit test. First, we assumed that the single instance can be clustered into any of the 3 PGs (PG0, PG1, and PG2) with equal probabilities. This means that we would expect to see predicted labels of the instance to be approximately 333 for all 3 PGs over the 1000 trials. Then, we compared the observed label counts to the expected label counts using the chi-square goodness of fit test. If the false-discovery rate *p*-value of this test is less than 0.05, we assume that the predictions are non-random. We then determined the predicted label of the instance to be the label with the highest standard residual. All of the 48 patients were classified into the 3 PGs (27 patients in PG0, 19 patients in PG1, and 2 patients in PG2).

## Results

### Multi-region sequencing allows the identification of patient-specific dysregulated genes

In this study, we performed RNA sequencing on multiple regions of the tumour and the adjacent normal from surgically resected HCC (shown in Fig. 1A). In a conventional all-patients differential expression (DE) analysis (all-patients analysis), tumour samples are grouped together and compared to the group of normal samples to identify tumour-related dysregulated genes. By design, genes that are consistently up- or down-regulated across samples – with distinct group mean values and small variance – get selected as differentially expressed between the two groups of samples. In our per-patient DE analysis (per-patient analysis), tumour samples from one patient are compared to the respective normal sample of the same patient, allowing the identification of dysregulated genes specific to the patient. Figure 1B summarizes the differences between the two approaches.

### Aggregation of per-patient analysis captures most conventional differential expression

Using conventional all-patients analysis, we obtained 2056 up-regulated and 4836 down-regulated genes in the tumour samples compared to the adjacent normal samples (Additional file 2). Gene set enrichment analysis (GSEA) of the up- and down-regulated genes showed enrichment of cell cycle-related gene sets among up-regulated genes, and enrichment of complement cascade-related gene sets among down-regulated genes (refer to Fig. S2A, Additional file 3).

In comparison, across the results of the per-patient analyses performed on 90 patients and using the same criteria to select differentially expressed genes, the average number of up-regulated genes in each patient was 1726 (standard deviation (s.d.) = 168). The average number of down-regulated genes was 1698 (s.d. = 980). About 90% (1851 out of 2056) up-regulated genes from the all-patients analysis were captured by the union set of up-regulated genes across the per-patient analyses. Similarly, 85% (4126 out of 4836) of the down-regulated genes from the all-patients analysis were captured by the union set of down-regulated genes across the per-patient analyses.

We defined the Differentially Expressed Patient Count (DEPC) for a gene as the number of patients in which the gene is up- or down-regulated based from per-patient analyses (up-regulated DEPC: Additional file 4, down-regulated DEPC table: Additional file 5). As all-patients analysis would capture genes consistently up- or down-regulated across the cohort, we expected the DE genes discovered in all-patients analysis to show high values of DEPC. To test this hypothesis, we divided DE genes from per-patient analyses into two groups: those also identified in the all-patients analysis (AP genes), and those not identified in all-patients analysis (NAP genes). Wilcoxon’s one-sided test reveals that in both up- and down-regulated cases, DEPC values of AP genes were significantly higher than NAP genes (DEPC-UP *p*-value < 2.2E-16; DEPC-DN *p*-value < 2.2E-16). This shows that indeed, all-patients analysis is limited to identifying dysregulated genes that are shared by large proportion of patients based on the much higher DEPC values of AP genes compared to NAP genes. The 205 up- and 710 down-regulated genes from the all-patients analysis that were missed by the per-patient analyses did now show any specific shared functions in GSEA analysis. The GSEA results excluding these genes also did not yield noticeably different results (refer to Fig. S2B).

To identify dysregulated genes in patient subgroups of different sizes, we used DEPC values as thresholds. A high DEPC threshold identifies dysregulated genes that are shared in large subgroups of patients, while a low DEPC threshold limits the identification to dysregulated genes shared in small subgroups of patients. A higher proportion of the high DEPC threshold genes would be part of the all-patient analysis, as those genes are commonly dysregulated across many of the patients. Indeed, the higher the DEPC threshold, the more of the up- and down-regulated genes from per-patient analyses were also picked up by all-patients analysis (shown in Fig. 1C). This was shown by the increasing proportion of the blue bars with increasing DEPC. Another interesting observation was that overall, there were higher numbers of NAP genes from up-regulated genes compared to the down-regulated genes.

We decided on a DEPC threshold value in order to identify dysregulated genes that were still part of the shared cancer gene modules, while retaining the patient specificity. We first computed the mean DEPC values for up-regulated and down-regulated genes separately. Setting a threshold of two standard deviations above the mean DEPC values led to the DEPC thresholds for the up-regulated and down-regulated genes of 27 and 34 respectively (shown in Fig. 1C). We obtained 887 up-regulated genes and 938 down-regulated genes from per-patient analyses above these thresholds. In these up-regulated genes, we observed that only 63.0% (559) of them were AP genes and 36.9% (328) were NAP genes (Fig. 1D). In contrast, most (99.9%) of the down-regulated genes with high DEPC were also discovered in the all-patients analysis. Our results show that per-patient analysis can detect genes that are up-regulated in a subgroup of patients yet missed by conventional all-patients analysis. Moreover, the results suggest that certain up-regulated HCC-related genes may be specific to subgroups of HCC patients, whereas most of the downregulated HCC-related genes are commonly shared across HCC patients. This is further supported by the larger proportion of AP genes from down-regulated genes than from the up-regulated genes in Fig. 1C, indicating that regardless of DEPC values, more of the down-regulated genes are commonly repressed genes across patient tumours.

### Conventional all-patients analysis omits HCC subgroup-specific genes due to high inter-individual variability

Conventional all-patients analysis missed some genes captured by aggregated per-patient analysis with high DEPC. We postulated that interpatient gene expression variability within normal samples may contribute to this difference. In all-patients analysis, genes with high inter-patient expression variability among normal samples would be less readily detected as differentially expressed. Those same genes may be captured, however, in per-patient analysis or aggregation of per-patient analysis to identify patient subgroups, indicating that these genes may still be potential cancer signature genes.

We investigated this in two ways. To assess whether he individuality of the samples indeed affects the differential expression testing, we generated density plots of the expression values of the top 50 AP genes and the top 50 NAP genes with the highest DEPC values (Fig. 1F). There was a clear distinction between the expression values of the top 50 AP genes in the tumour and adjacent normal samples (Fig. 1F, top). In contrast, the top 50 NAP genes showed highly heterogeneous baseline normal expression, without any clear distinction between the tumour and adjacent normal samples (Fig. 1F, bottom). This observation was further supported by the 387 NAP genes showing significantly higher standard deviations of expression values in the normal samples, compared to those of the 559 AP genes (*p*-value < 2.2E-16; Fig. 1E). These results suggest that the high inter-individual variability may be a contributing factor to why these genes were omitted by the conventional analysis even though these genes were up-regulated in more than 30% of the patients.

We also tested whether the per-patient analysis was dependent on the correct pairing between normal and tumour samples. Because per-patient analysis uses fewer samples than all-patient analysis, and because each per-patient analysis is limited to a single adjacent normal sample, it is conceivable that the per-patient analysis generates artifacts. We wanted to ensure that the patient subgroups were not just artefacts of the sample size limitations. We repeated the per-patient and DEPC analysis with 100 random permutations of adjacent normal sample labels (Fig. S1). The correct pairing identified more dysregulated genes unique and specific to each patient (and shared between less than 5 patients). Larger DEPC values of permuted pairings picked up more dysregulation, and the permuted analysis began to resemble the all-patients analysis, in which samples were not paired.

We then examined three known cancer-associated genes – CDK1, E2F3, and CD24 – from the 887 genes (Fig. 1G). CDK1 is a cell-cycle gene that is up-regulated in HCC tumours [27] and was one of the AP genes in our analysis. CDK1 was consistently up-regulated across most of the patients, with a clear distinction between the expression values in tumour and adjacent normal samples (Fig. 1G, top). On the other hand, E2F3 and CD24, which are also commonly dysregulated in cancer [28, 29] were classified as NAP genes in our analysis as they were up-regulated in at least 30% of the patients but were not identified by the all-patients analysis. The expression values of these two genes showed that indeed, even though some patients’ samples showed a clear distinction between normal and adjacent tumour samples, the dysregulation pattern was not shared across all patients (Fig. 1G, middle and bottom). Furthermore, even though E2F3 and CD24 were up-regulated in some patients, other patients showed down-regulation of these genes. They are examples of cancer-associated genes that exhibit high levels of patient and subgroup specificity, which our per-patient analyses managed to identify.

### Patient-specific differentially expressed genes with high DEPC scores are associated with metabolism, proliferation, and known cancer modules

To understand the known functions and mechanisms of the patient subgroup-specific differentially expressed genes, we performed GSEA on the 328 NAP genes against two groups of gene sets, namely the gene sets from REACTOME and CGP sets from MSigDB (Fig. 1H, Additional file 6) [30]. Many of the enriched REACTOME gene sets (Fig. 1H, left) were related to lipid and fatty acid metabolism, as well as cholesterol homeostasis. The results indicate that a subset of patients’ tumours showed significant changes in the metabolism of lipids, possibly reflecting the metabolic variability among the patients’ liver tissues. The results also hinted at subgroup-specific activation of cancer pathways, evident from many of NAP genes belonging to WNT signalling and proliferation gene sets (Fig. 1H, left). The GSEA results against the CGP gene sets also revealed that there are significant overlaps between these NAP genes and curated liver cancer genes (Fig. 1H, right). These results indicate that the differential activation of these NAP genes across the patients might be useful in identification of HCC subgroups.

### Patient-specific transcriptomic profiles reveal novel HCC patient subgroups with a strong correlation to recurrence

Based on the earlier observations, we hypothesized that the pattern of patient-specific gene expression changes could be useful in identifying patient subgroups associated with different recurrence trajectories. Unlike conventional cancer subtyping analysis which identifies patients with relatively high/low expression of some cancer signature genes, we shifted the focus to whether or not the subgroup-specific genes were dysregulated in a patient’s tumour compared to the patient’s normal tissue.

Using the 328 NAP genes, we first generated the fold-change matrix based on the per-patient analyses results (Fig. 2A, right). We further simplified the matrix and generated the categorical matrix with 3 values – -1, 0, or 1 – representing the down-regulation, no change, or up-regulation of the gene in the tumour samples compared to the patient's normal samples respectively (Fig. 2B, left). Some noticeable patterns of gene activation in subgroups of patients emerged (Fig. 2B, left), which were not obvious with the starting normalized gene count matrix (Fig. 2A, left). The categorical data were then subjected to co-clustering analysis, producing 3 patient groups (PGs) (Fig. 2B, right).

We investigated the patient groups produced by the co-clustering analysis (42 patients in PG0, 36 patients in PG1, and 12 patients in PG2). We focused on PG0 and PG1 since patients in PG2 mostly showed no changes across all genes. Patients in PG1 have significantly more up-regulated genes than patients in PG0 (*p*-value: < 2.2E-16), suggesting that patients in PG1 generally show more up-regulation in these subgroup-specific DE genes than patients in PG0.

By performing Kaplan Meier survival analysis, we observed that patients in PG1 have shorter recurrence-free survival after surgical resection compared to patients in PG0 (*p*-value < 0.05) (Fig. 2C, left). This supports our hypothesis that patient stratification based on the patient subgroup-specific activated genes yields clinically relevant grouping that was not detectable from the absolute level of expression values. Subsequently, we examined if the patients in PG0 and PG1 were different in terms of their clinical profiles (Table 1). The clustering in PG0 and PG1 showed significant association with sex, the degree of fibrosis, chronic hepatitis B status, prothrombin time and alpha fetoprotein levels (AFP). Patients in PG1 also have significantly higher prothrombin time reflecting their poorer liver function, and higher level of AFP reflecting poor tumour differentiation, than patients in PG0. Fig. S3 showed that none of these variables, by themselves, had any effect on the differential prognosis we observed between the two PGs. This suggests that the patient grouping we obtained can separate the patients into the two differential prognosis groups and is independent of the clinical variables we obtained.

**Table 1:** Chi-square/Fisher’s exact/Wilcoxon test of clinical variables against the patient clusters PG0 and PG1

Variable		PG0	PG1	*P*-value	significance
**N**		42	36		
**Sex**	Female	4	10	0.043	*
	Male	38	26		
**Ethnicity**	Chinese	31	20	0.363	
	Filipino	3	1		
	Indian	1	2		
	Indonesian	1	1		
	Malay	2	2		
	Thai	2	7		
	Others	2	3		
**Significant Alcohol Consumption**	Yes	11	9	0.683	
	No	23	17		
	Unknown	8	10		
**Child's Pugh score**	A	41	36	1.000	
	B	1	0		
**Diabetes**	Yes	17	12	0.636	
	No	25	24		
**Tumour Multiplicity**	Yes	7	8	0.588	
	No	35	28		
**Fibrosis Stage**	0	14	6	0.010^*^	**
	1	3	4		
	2	1	9		
	3	11	4		
	4	9	11		
**Microvascular Invasion**	Yes	12	17	0.110	
	No	30	19		
**Edmondson Grade**	1	5	2	0.058	
	2	26	15		
	3	11	17		
	4	0	2		
**Steatosis**	0–5%	20	23	0.268	
	5–33%	14	11		
	33–66%	3	0		
**Overall Survival**	Alive	36	25	0.110	
	Dead	6	11		
**Tumour Stage TNM V8**	I	25	17	0.216	
	II	13	10		
	III	4	9		
**Recurrence status**	Yes	24	14	0.125	
	No	18	22		
**HBV Status**	positive	23	29	0.030	*
	negative	19	7		
**HCV Status**	positive	4	2	0.681	
	negative	38	34		
**Max. Tumour Diameter (cm)**		6.84 ± 4.94	6.35 ± 3.93	0.876	
**Albumin (g/L)**		40.95 ± 4.32	41.3 ± 3.55	0.751	
**Bilirubin (umol/L)**		13.73 ± 4.57	13.12 ± 5.68	0.348	
**AST (U/L)**		50.65 ± 36.2	50.23 ± 52.29	0.854	
**ALT (U/L)**		50.47 ± 53.97	33.86 ± 19.92	0.196	
**Alkaline Phosphatase (U/L)**		108.85 ± 53.44	125.41 ± 121.7	0.943	
**Prothrombin Time (secs)**		10.91 ± 0.96	11.55 ± 1.35	0.025	*
**Platelets (× 10^9)**		232.82 ± 89.59	239.14 ± 71.48	0.344	
**AFP (ng/ml)**		1925.1 ± 9012.17	4362.23 ± 11,826.79	0.023	*
**Recurrence-free survival days**		675.76 ± 462.37	521.72 ± 433.32	0.132	

^*^For fibrosis stage, a post hoc Fisher’s exact with Bonferroni correction was performed. Only stage 2 vs stage 3 was significant with *p*-adjusted value of 0.036

To further validate the prognostic value of the patient subgroups, we obtained RNA-seq data from TCGA-LIHC and utilized a machine learning approach, as seen in Fig. S4A, to stratify the patients into their respective PGs. Machine learning was used because of the lack of biological replicates for the TCGA patients, and to utilize the paired sample gene counts instead. Across the 1000 trials from the data, the machine learning model achieved a mean accuracy of 90.73% with a standard deviation of 3.44% (Fig. S4B). Survival analysis reveals that the TCGA-LIHC patients classified into PG1 are more likely to recur than the TCGA-LIHC patients classified into PG0 (*p*-value < 0.05) (Fig. 2C, right). Therefore, this supports the results we obtained from our data and strengthened the evidence that these subgroup-specific activated genes are useful for patient stratification.

### Overall differences in the expression profiles between the patient groups with different recurrence rates

**Fig. 3:**
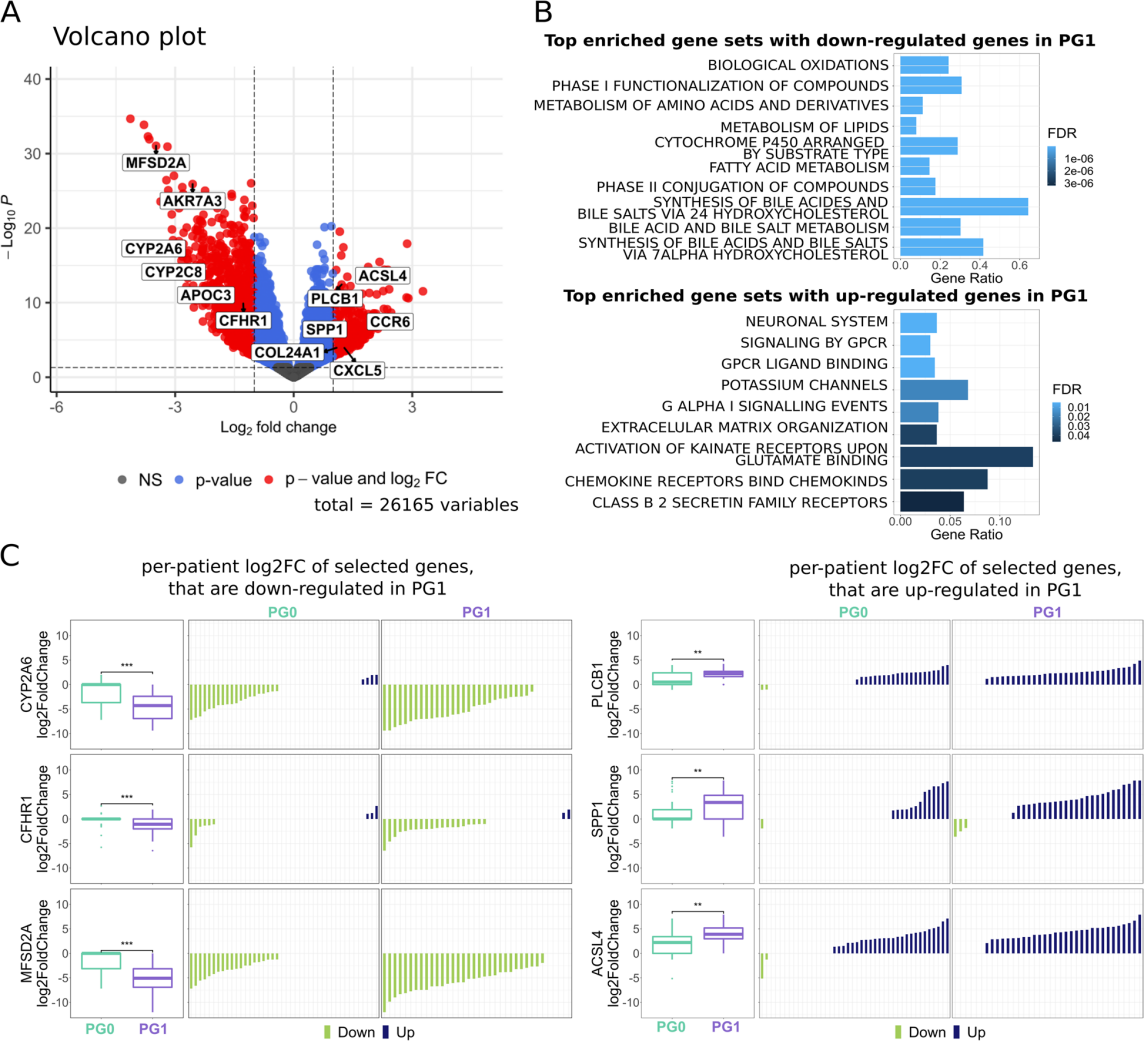
Transcriptomic differences between PG0 and PG1 patients’ tumour tissues. **A**: Volcano plot of log2FoldChange values against -log adjusted *p*-value of differentially expressed genes between patients in PG0 and PG1 (adjusted *p*-value cut-off: 0.05, log2FoldChange absolute cut-off: 1). **B**: Top 10 enriched gene sets with genes that are down- regulated (top)/ up-regulated (bottom) in PG1. **C:** Log2FoldChange plots from per-patient analysis with example genes that are down-regulated (left)/ up-regulated (right) in PG1. Log2FoldChange barplots are ordered by ascending order for each gene (patient orders are not the same across different example genes)

Comparing the RNA-seq data of the tumour samples between PG0 and PG1 revealed that there were 863 down-regulated and 377 up-regulated genes in PG1 patients compared to the PG0 patients (Additional file 7). There were not only more down-regulated genes than up-regulated genes in PG1, but also a higher degree of dysregulation in the down-regulated genes than the up-regulated genes (Fig. 3A). We selected a few example genes differentially expressed between the two patient groups and compared them to the log2FoldChange values from the per-patient analyses (Fig. 3C). These plots showed significant differences in the log2FoldChange values between patients in PG0 and PG1, with the overall gene expression changes broadly in line with the DE analysis.

The down-regulated genes showed significant overlaps with the lipid metabolism related and complement cascade gene sets (Fig. 3B, top, Additional file 8). Since the patients in PG1 show less activation in these genes than patients in PG0, the down-regulation of these genes be associated with recurrence. This finding is in keeping with the literature on known HCC prognostic markers. Low expression of lipid metabolism-related genes such as APOC3, CYP2A6, and CYP2C8 were indeed associated with lower recurrence-free survival (RFS) in HCC [31, 32]. Similarly, the repression of the complement cascade-related factors such as CFHR1 was associated with worse RFS [33]. We also investigated specific genes, such as MFSD2A and AKR7A3, that showed high degree of down-regulation in PG1 patients compared to the PG0 patients (Fig. 3A). The down-regulation of these genes has been linked to poorer survival [34, 35] and our results here suggests that they may also be linked to poor RFS.

The up-regulated genes in PG1 patients were largely related to the neuronal system, GPCR signalling, and extracellular matrix organisation (Fig. 3B, bottom, Additional file 8). We validated these results against current literature. For example, high expression of PLCB1, a gene that is in both the neuronal system gene set and GPCR signalling gene set, has been reported to be associated with poorer RFS [36]. Other example genes such as CCR6 and CXCL5, which are involved in the GPCR signalling gene set, are also associated with poorer RFS [37, 38]. In the extracellular matrix organisation gene set, the high expression of two example genes such as COL24A1 and SPP1 have been shown to be associated with higher recurrence rate [39, 40].

Lastly, we observed another example gene, ACSL4, which has garnered a lot of attention in the field of HCC in recent years. ACSL4, long chain acyl-CoA synthetase, has been shown to be able to stabilize c-Myc expression and promote tumour cell proliferation and tumour progression in HCC, and patients with high expression of ACSL4 have poorer RFS [41]. Additionally, ACSL4 has also been proposed to be a predictive biomarker of sorafenib sensitivity for HCC patients [42]. This suggests that the patient grouping from this analysis may have translational value in predicting sorafenib sensitivity. Overall, the above results reaffirm that our patient subgrouping indeed captures known, as well as potentially novel, prognostic markers.

### Patient-specific gene dysregulation may explain the low response rates of current HCC therapeutic treatments

Another advantage of the per-patient analysis is that we can define the proportionality of activation/repression of specific genes across the patients. This is an important feature when considering the marginal BORR of current systemic therapies for unresectable HCC. That only a limited proportion of patients showed dysregulation in purported drug target genes may help understand the low drug efficacy. Here, we focus on tyrosine-kinase receptors inhibitors: the two most common first-line drugs (Sorafenib, Lenvatinib) and two second-line drugs (Ramucirumab, Cabozantinib).

**Fig. 4:**
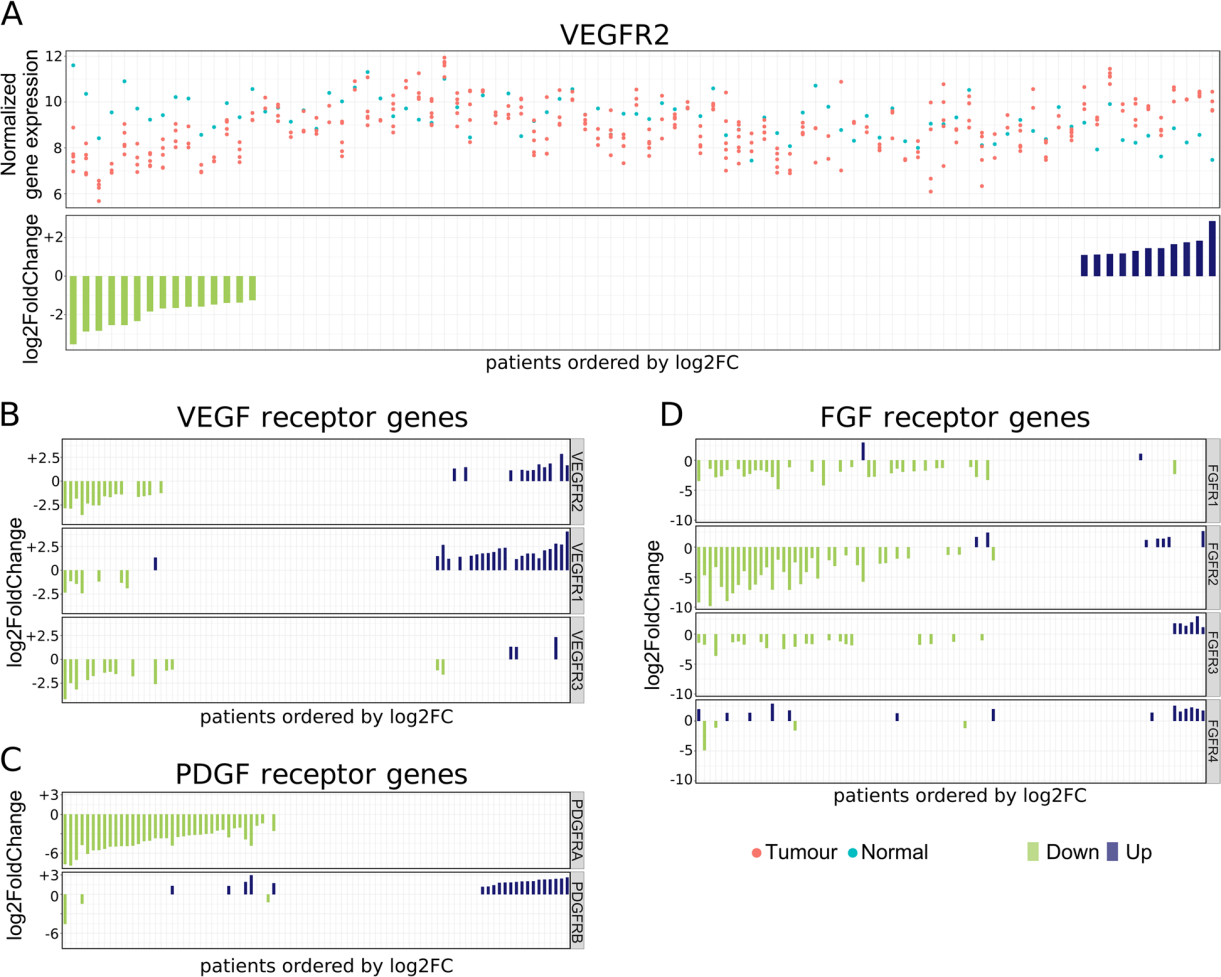
Many HCC drug target tyrosine kinases receptors show variable expression changes across different patients. **A**: Normalized gene expression and their respective log2FoldChange values from per-patient analysis of VEGFR2. **B-D**: Log2FoldChange barplots from per-patient analysis of VEGFRs (**B**), PDGFRs (**C**) and FGFRs (**D**). Patient orders are not the same across **A**, **B**, **C** and **D**

Across these 4 drugs, the BORR ranges from 1–24%, with most of the clinical trials showing that BORR of these drugs are less than 10% [3]. Since these drugs only work for a small proportion of the HCC patients, we investigated the patient-specific dysregulation of the drugs’ targets. The common and main target of these 4 drugs is the vascular endothelial growth factor receptor 2 (VEGFR2) [43–46]. However, in our patient-specific transcriptomic profiles, we found that only 12% of the patients showed upregulation in VEGFR2 and 16% of the patients displayed down-regulation in VEGFR2 (Fig. 4A, Table 2). Since these drugs inhibit VEGFR2, they may have limited efficacy when VEGFR2 is not overexpressed in the tumours. The small proportion of patients with overexpressed VEGFR2 may explain the low response rate of these drugs.

**Table 2:** Per-patient results for target genes of Sorafenib, Lenvatinib, Cabozantinib, and Ramucirumab. ([43–46])

Gene	Down-regulated DEPC	Up-regulated DEPC	Down-regulated in all-patients analysis	Up-regulated in all-patients analysis	Targeted by
VEGFR2/KDR	15	11	No	No	Sorafenib, Lenvatinib, Ramucirumab, Cabozantinib
VEGFR1/FLT1	7	22	No	No	Sorafenib, Lenvatinib, Cabozantinib
VEGFR3/FLT4	14	3	No	No	Sorafenib, Lenvatinib, Cabozantinib
FLT3	11	5	Yes	No	Sorafenib
PDGFRA	37	0	Yes	No	Lenvatinib
PDGFRB	3	21	No	No	Sorafenib
FGFR1	31	2	Yes	No	Lenvatinib
FGFR2	34	7	Yes	No	Lenvatinib
FGFR3	20	6	No	No	Lenvatinib
FGFR4	4	14	No	No	Lenvatinib
KIT	1	9	No	No	Sorafenib, Lenvatinib
RET	28	2	Yes	No	Sorafenib, Lenvatinib, Cabozantinib
AXL	32	0	Yes	No	Cabozantinib
MET	3	10	No	No	Cabozantinib
BRAF	1	5	No	No	Sorafenib
RAF1	1	1	No	No	Sorafenib

Other families of tyrosine kinase receptors that are HCC drug targets include platelet-derived growth factor receptors (PDGFRs), and fibroblast growth factor receptors (FGFRs) [43–46]. We observed that these receptors are only overexpressed in small proportions of patients ranging from 2% in FGFR1 to 24% in VEGFR1 (Fig. 4B-D, Table 2). Some of these receptors are under-expressed in a number of patients. Notably, our all-patients analysis showed down-regulation of PDGFRA, FGFR1 and FGFR2. The low proportion of patients showing overexpression of these receptors is in keeping with the poor BORRs of these current HCC drugs.

## Discussion/conclusion

The ability to derive patient-specific differential transcriptomic profiles is a powerful tool. While conventional DE analysis is useful in the overall description of the changing transcriptomic landscape, it lacks the granularity to identify patient and subgroup-specific dysregulated genes. In this study, we show that patient innate differences at the normal baseline hinders the conventional approach from picking up patient-specific DE genes. Our results highlight the importance of considering the variability in patients’ normal tissues, while emphasising the value of using multi-region samples and per-patient analysis. With this analysis, we can derive patient-specific DE genes and their respective transcriptomic profiles. Although the patient-specific transcriptomic profiles may have limited immediate impact in understanding the tumour biology of HCC, they are imperative to the development of personalized cancer therapeutics.

Aggregating the results of patient-specific DE genes also enables us to identify subgroup-specific DE genes, which are missed out by the conventional analysis. Referring the subgroup-specific DE genes to the CGP gene sets from MSigDB, we discovered that these genes have significant overlap with 2 gene sets [10, 47], which describe a stem-cell HCC subtype and a proliferation subclass respectively. This suggests that the subgroups we derived may be linked to these subclasses. We also observed that patients in PG1, which have more up-regulated proliferation-related genes, have elevated AFP compared to patients in PG0. This is in line with the result of Chiang et al. [10], where they also showed higher AFP in the proliferation subclass. The significant overlap of our subgroup-specific genes between these two gene sets suggests that our study and the two studies are describing the same subclass (PG1). However, our analysis provides a more comprehensive picture of this proliferation-related subclass. Both studies rely on specific genes or molecular signatures and mutational profiles to determine the subclass. In contrast, our data-driven stratification is not limited to conventional sets of molecular signatures and is free from literature bias. Moreover, our stratification strategy is much more flexible and may yield different results if we were to modify the DEPC threshold.

We were able to link differential prognosis to the newly discovered subgroups and observe similar results using a machine-learning approach on TCGA-LIHC data. This minimizes any potential bias that may be present in our dataset. The clinical correlation with disease recurrence presented in both datasets validates our unique approach to identify HCC subgroups. The DE analysis between PG0 and PG1 identified multiple prognostic markers that are supported by the current literature. This proves that our methodology is valuable in identifying HCC prognostic subgroups that relies on either clinical parameters, histological and immunological hallmarks, or specific gene signatures and biomarkers. These conventional approaches only capture a narrow perspective of the tumour biology with selective indicators whereas our method shows a much wider range of known and potential indicators that can help to elucidate HCC progression.

The per-patient analysis also provides insights into the poor BORRs of current targeted agents used in HCC. We showed that the target genes of current agents are only overexpressed in a limited proportion of patients, which may explain why the response to treatment is also limited to a subset of patients: patients whose tumours lack overexpression or show under-expression of these targets may not be appropriate for these agents. We wish to highlight that the dysregulation may differ between different receptors: a patient may show, for example, down-regulation in VEGFRs but up-regulation in FGFRs. Our results reaffirm the complexity of the landscape of tyrosine kinase receptors dysregulation in HCC. Since the majority of the patients showed no significant dysregulation of these receptor genes, these patients may be more suited for non-tyrosine kinase-based therapy options.

In this paper, we developed a novel analysis pipeline that expands from the conventional norm of DE analysis and showed that by leveraging multi-region sampling, patient-specific analysis confers a better ability to identify subgroup-specific gene dysregulation and subsequent patient stratification with prognostic value. Additionally, the patient-specific analysis provides a potential framework for understanding poor BORRs of current HCC systemic therapies. This patient-specific approach will serve as a steppingstone to help researchers understand the molecular subtypes in HCC and their clinical trajectories, and aid clinicians as they pivot towards precision oncology and personalized cancer therapeutics.

## Supplementary Information

**Additional file 1: Supplementary Table S1.** Sample information. Tumor and adjacent normal tissues collected from 90 HCC patients were used in this study. Among the 90 patients, there were 17 patients with multifocal HCC. Only 31 tumor tissue samples (from 6 patients) were collected from multiple nodules.

**Additional file 2: Supplementary Table S2.** List of up-regulated genes in the tumour samples compared to the adjacent normal samples from all-patients analysis.** Supplementary Table S3.** List of down-regulated genes in the tumour samples compared to the adjacent normal samples from all-patients analysis.

**Additional file 3: Supplementary Table S4.** Top 20 gene sets from GSEA of up-regulated and down-regulated genes in all-patients analysis.

**Additional file 4: Supplementary Table S5.** DEPC values for up-regulated genes from per-patient analyses.

**Additional file 5: Supplementary Table S6.** DEPC values for down-regulated genes from per-patient analyses.

**Additional file 6: Supplementary Table S7.** Top 20 REACTOME and CGP gene sets from GSEA of 328 NAP genes.

**Additional file 7: Supplementary Table S8.** List of down-regulated genes in PG1 patients in comparison to PG0.** Supplementary Table S9.** List of up-regulated genes in PG1 patients in comparison to PG0.

**Additional file 8: Supplementary Table S10.** Top 20 REACTOME gene sets from GSEA of down-regulated and up-regulated genes in PG1.

**Additional file 9: Fig. S1.** Number of up-regulated (top) and down-regulated (bottom) genes per each DEPC value, using randomly assigned adj.normal samples. Coloured lines represent mean±s.d. after 100 iterations. Black line represents the values with the original N-T pairing for each patient.

**Additional file 10: Fig. S2.** **A**. GSEA results for up-regulated genes in tumour tissues from all-patients (AP) analysis. **B**: GSEA results for down-regulated genes in tumour tissues from AP analysis. Across both panels, top chart shows results including all the up-regulated genes from the AP analysis, while the bottom chart excludes the genes also detected in PP analyses.

**Additional file 11: Fig. S3.** Kaplan-Meier recurrence-free survival plots for patients that are categorized based on sex(top), fibrosis stages (middle), and HBV status (bottom).

**Additional file 12: Fig. S4.** **A**. Schematic illustration of machine-learning based patient stratification strategy for TCGA- LIHC paired sample data. **B**: Patient stratification classification accuracies of PG0, PG1, PG2 and combined (Total).

## Data Availability

The datasets generated during the current study are available in the European Genome-Phenome Archive (EGA) with accession number EGAD00001009042 (https://ega-archive.org/datasets/EGAD00001009042).

## Abbreviations

AFP: Alpha fetoprotein levels
BORR: Best overall response rates
CPM: Counts-per-million
DE: Differentially expressed
DEPC: Differentially Expressed Patient Count
FGFRs: Fibroblast growth factor receptors
GSEA: Gene set enrichment analysis
HCC: Hepatocellular carcinoma
H&E: Haematoxylin & eosin
METAVIR: Meta-analysis of histological data in viral hepatitis
MLP: Multi-layer perceptron
PDGFRs: Platelet-derived growth factor receptors
PFI: Progression-free interval
RFS: Recurrence-free survival
SGD: Stochastic gradient descent
s.d.: Standard deviation
TCGA-LIHC: The Cancer Genome Atlas-Liver Hepatocellular Carcinoma
TMM: Trimmed Mean of *M*-values
TND: Tumour-Normal-Difference
VEGFR2: Vascular endothelial growth factor receptor 2
