# Supplementary material for: Multi-region sampling with paired sample sequencing analyses reveals sub-groups of patients with novel patient-specific dysregulation in Hepatocellular Carcinoma
Source: BMC Cancer. 2023 Feb 3;23:118. doi: 10.1186/s12885-022-10444-3 (PMC9896715; doi:10.1186/s12885-022-10444-3)
Supplement: Supplementary file 3 — Additional file 3: Supplementary Table S4. Top 20 gene sets from GSEA of up-regulated and down-regulated genes in all-patients analysis. [file 12885_2022_10444_MOESM3_ESM.docx]

**Supplementary Table S4. Top 20 gene sets from GSEA of up-regulated and down-regulated genes in all-patients analysis**

| Gene set | Overlap | Size | pval | FDR |
| --- | --- | --- | --- | --- |
| **Top 20 gene sets from GSEA of up-regulated genes in all-patients analysis** |  |  |  |  |
| CELL_CYCLE | 157 | 693 | 7.81E-58 | 1.25E-54 |
| CELL_CYCLE_MITOTIC | 132 | 561 | 1.10E-50 | 8.80E-48 |
| CELL_CYCLE_CHECKPOINTS | 90 | 292 | 3.51E-45 | 1.88E-42 |
| M_PHASE | 83 | 416 | 6.44E-27 | 2.58E-24 |
| RHO_GTPASE_EFFECTORS | 71 | 324 | 9.17E-26 | 2.94E-23 |
| RESOLUTION_OF_SISTER_CHROMATID_COHESION | 44 | 126 | 2.71E-25 | 7.24E-23 |
| MITOTIC_SPINDLE_CHECKPOINT | 40 | 111 | 1.02E-23 | 2.33E-21 |
| DEPOSITION_OF_NEW_CENPA_CONTAINING_NUCLEOSOMES_AT_THE_CENTROMERE | 32 | 73 | 2.29E-22 | 4.59E-20 |
| MITOTIC_PROMETAPHASE | 51 | 202 | 8.38E-22 | 1.49E-19 |
| G2_M_CHECKPOINTS | 46 | 168 | 2.26E-21 | 3.62E-19 |
| CHROMOSOME_MAINTENANCE | 42 | 140 | 2.64E-21 | 3.84E-19 |
| MEIOTIC_RECOMBINATION | 33 | 87 | 1.24E-20 | 1.66E-18 |
| RHO_GTPASES_ACTIVATE_FORMINS | 41 | 140 | 2.13E-20 | 2.63E-18 |
| MEIOSIS | 36 | 119 | 1.33E-18 | 1.52E-16 |
| SEPARATION_OF_SISTER_CHROMATIDS | 45 | 191 | 3.85E-18 | 4.11E-16 |
| CONDENSATION_OF_PROPHASE_CHROMOSOMES | 28 | 74 | 1.09E-17 | 1.09E-15 |
| REPRODUCTION | 38 | 145 | 3.32E-17 | 2.83E-15 |
| MITOTIC_METAPHASE_AND_ANAPHASE | 49 | 236 | 3.35E-17 | 2.83E-15 |
| DNA_METHYLATION | 26 | 65 | 3.10E-17 | 2.83E-15 |
| DISEASES_OF_PROGRAMMED_CELL_DEATH | 32 | 103 | 4.47E-17 | 3.58E-15 |
| **Top 20 gene sets from GSEA of down-regulated genes in all-patients analysis** |  |  |  |  |
| COMPLEMENT_CASCADE | 99 | 114 | 8.93E-75 | 1.43E-71 |
| CREATION_OF_C4_AND_C2_ACTIVATORS | 68 | 71 | 6.16E-59 | 4.94E-56 |
| INITIAL_TRIGGERING_OF_COMPLEMENT | 72 | 79 | 3.74E-58 | 2.00E-55 |
| SCAVENGING_OF_HEME_FROM_PLASMA | 66 | 69 | 4.02E-57 | 1.61E-54 |
| BINDING_AND_UPTAKE_OF_LIGANDS_BY_SCAVENGER_RECEPTORS | 74 | 98 | 1.01E-47 | 3.23E-45 |
| IMMUNOREGULATORY_INTERACTIONS_BETWEEN_A_LYMPHOID_AND_A_NON_LYMPHOID_CELL | 105 | 188 | 1.57E-47 | 4.20E-45 |
| CD22_MEDIATED_BCR_REGULATION | 56 | 61 | 6.45E-46 | 1.48E-43 |
| FCGR_ACTIVATION | 60 | 69 | 7.43E-46 | 1.49E-43 |
| FCGR3A_MEDIATED_IL10_SYNTHESIS | 65 | 95 | 1.07E-37 | 1.91E-35 |
| ROLE_OF_PHOSPHOLIPIDS_IN_PHAGOCYTOSIS | 60 | 82 | 1.33E-37 | 2.13E-35 |
| ANTIGEN_ACTIVATES_B_CELL_RECEPTOR_BCR_LEADING_TO_GENERATION_OF_SECOND_MESSENGERS | 61 | 86 | 6.57E-37 | 9.58E-35 |
| ROLE_OF_LAT2_NTAL_LAB_ON_CALCIUM_MOBILIZATION | 55 | 71 | 7.48E-37 | 9.99E-35 |
| ANTI_INFLAMMATORY_RESPONSE_FAVOURING_LEISHMANIA_PARASITE_INFECTION | 97 | 224 | 5.48E-32 | 6.76E-30 |
| FCERI_MEDIATED_MAPK_ACTIVATION | 57 | 87 | 1.14E-31 | 1.30E-29 |
| HEMOSTASIS | 192 | 678 | 5.69E-31 | 6.09E-29 |
| FCERI_MEDIATED_CA_2_MOBILIZATION | 56 | 86 | 6.28E-31 | 6.29E-29 |
| INNATE_IMMUNE_SYSTEM | 267 | 1106 | 5.70E-30 | 5.38E-28 |
| BIOLOGICAL_OXIDATIONS | 92 | 219 | 3.43E-29 | 3.06E-27 |
| PARASITE_INFECTION | 63 | 116 | 3.73E-28 | 3.15E-26 |
| LEISHMANIA_INFECTION | 110 | 309 | 3.53E-27 | 2.83E-25 |
