# Supplementary material for: Multi-region sampling with paired sample sequencing analyses reveals sub-groups of patients with novel patient-specific dysregulation in Hepatocellular Carcinoma
Source: BMC Cancer. 2023 Feb 3;23:118. doi: 10.1186/s12885-022-10444-3 (PMC9896715; doi:10.1186/s12885-022-10444-3)
Supplement: Supplementary file 6 — Additional file 6: Supplementary Table S7. Top 20 REACTOME and CGP gene sets from GSEA of 328 NAP genes. [file 12885_2022_10444_MOESM6_ESM.docx]

**Supplementary Table S7. Top 20 REACTOME and CGP gene sets from GSEA of 328 NAP genes**

| Gene set | Overlap | Size | pval | FDR |
| --- | --- | --- | --- | --- |
| **Top 20 REACTOME gene sets from GSEA of 328 NAP genes** |  |  |  |  |
| METABOLISM_OF_LIPIDS | 28 | 742 | 2.48E-11 | 3.99E-08 |
| LINOLEIC_ACID_LA_METABOLISM | 5 | 8 | 1.90E-09 | 1.52E-06 |
| FATTY_ACID_METABOLISM | 13 | 178 | 3.00E-09 | 1.60E-06 |
| FATTY_ACYL_COA_BIOSYNTHESIS | 7 | 37 | 1.85E-08 | 7.41E-06 |
| ALPHA_LINOLENIC_OMEGA3_AND_LINOLEIC_OMEGA6_ACID_METABOLISM | 5 | 13 | 4.22E-08 | 1.35E-05 |
| TCF_DEPENDENT_SIGNALING_IN_RESPONSE_TO_WNT | 12 | 234 | 5.82E-07 | 0.000156 |
| ACTIVATION_OF_GENE_EXPRESSION_BY_SREBF_SREBP | 6 | 42 | 1.14E-06 | 0.00026 |
| CONDENSATION_OF_PROPHASE_CHROMOSOMES | 7 | 74 | 2.50E-06 | 0.000501 |
| MITOTIC_PROPHASE | 9 | 143 | 2.87E-06 | 0.000512 |
| METABOLISM_OF_STEROIDS | 9 | 151 | 4.49E-06 | 0.000721 |
| REGULATION_OF_CHOLESTEROL_BIOSYNTHESIS_BY_SREBP_SREBF | 6 | 55 | 5.74E-06 | 0.000838 |
| SIGNALING_BY_WNT | 12 | 331 | 2.02E-05 | 0.002699 |
| CHREBP_ACTIVATES_METABOLIC_GENE_EXPRESSION | 3 | 8 | 2.90E-05 | 0.003191 |
| PRC2_METHYLATES_HISTONES_AND_DNA | 6 | 73 | 2.98E-05 | 0.003191 |
| TRANSCRIPTIONAL_REGULATION_BY_SMALL_RNAS | 7 | 107 | 2.88E-05 | 0.003191 |
| CELLULAR_SENESCENCE | 9 | 198 | 3.91E-05 | 0.003693 |
| ERCC6_CSB_AND_EHMT2_G9A_POSITIVELY_REGULATE_RRNA_EXPRESSION | 6 | 76 | 3.75E-05 | 0.003693 |
| PROTEIN_UBIQUITINATION | 6 | 79 | 4.68E-05 | 0.004168 |
| DNA_DAMAGE_TELOMERE_STRESS_INDUCED_SENESCENCE | 6 | 80 | 5.02E-05 | 0.00424 |
| CELL_CYCLE_MITOTIC | 15 | 561 | 6.68E-05 | 0.005355 |
| **Top 20 CGP gene sets from GSEA of 328 NAP genes** |  |  |  |  |
| PATIL_LIVER_CANCER | 90 | 657 | 1.98E-83 | 6.65E-80 |
| CAIRO_HEPATOBLASTOMA_UP | 35 | 214 | 5.95E-35 | 1.00E-31 |
| LIAO_METASTASIS | 47 | 542 | 1.02E-33 | 1.15E-30 |
| ACEVEDO_LIVER_CANCER_UP | 58 | 972 | 1.00E-32 | 8.44E-30 |
| ACEVEDO_LIVER_TUMOR_VS_NORMAL_ADJACENT_TISSUE_UP | 49 | 863 | 1.07E-26 | 7.24E-24 |
| DESERT_STEM_CELL_HEPATOCELLULAR_CARCINOMA_SUBCLASS_UP | 27 | 242 | 1.27E-22 | 7.12E-20 |
| CHIANG_LIVER_CANCER_SUBCLASS_PROLIFERATION_UP | 20 | 178 | 3.92E-17 | 1.89E-14 |
| DODD_NASOPHARYNGEAL_CARCINOMA_DN | 48 | 1408 | 5.40E-17 | 2.28E-14 |
| ONKEN_UVEAL_MELANOMA_UP | 35 | 786 | 5.20E-16 | 1.95E-13 |
| CHICAS_RB1_TARGETS_CONFLUENT | 30 | 566 | 7.33E-16 | 2.47E-13 |
| SMID_BREAST_CANCER_BASAL_DN | 30 | 695 | 1.58E-13 | 4.83E-11 |
| CAIRO_HEPATOBLASTOMA_CLASSES_UP | 28 | 612 | 2.61E-13 | 7.31E-11 |
| NUYTTEN_EZH2_TARGETS_DN | 35 | 1018 | 9.96E-13 | 2.58E-10 |
| BORCZUK_MALIGNANT_MESOTHELIOMA_UP | 20 | 311 | 1.69E-12 | 4.07E-10 |
| VECCHI_GASTRIC_CANCER_EARLY_UP | 23 | 437 | 2.24E-12 | 5.04E-10 |
| GOZGIT_ESR1_TARGETS_DN | 30 | 780 | 2.91E-12 | 6.12E-10 |
| WANG_CISPLATIN_RESPONSE_AND_XPC_UP | 16 | 185 | 3.42E-12 | 6.78E-10 |
| NIKOLSKY_BREAST_CANCER_8Q12_Q22_AMPLICON | 14 | 131 | 4.40E-12 | 8.23E-10 |
| LINDGREN_BLADDER_CANCER_CLUSTER_1_DN | 21 | 380 | 8.33E-12 | 1.48E-09 |
| FOURNIER_ACINAR_DEVELOPMENT_LATE_2 | 18 | 280 | 2.24E-11 | 3.78E-09 |
