# Supplementary material for: Multi-region sampling with paired sample sequencing analyses reveals sub-groups of patients with novel patient-specific dysregulation in Hepatocellular Carcinoma
Source: BMC Cancer. 2023 Feb 3;23:118. doi: 10.1186/s12885-022-10444-3 (PMC9896715; doi:10.1186/s12885-022-10444-3)
Supplement: Supplementary file 8 — Additional file 8: Supplementary Table S10. Top 20 REACTOME gene sets from GSEA of down-regulated and up-regulated genes in PG1. [file 12885_2022_10444_MOESM8_ESM.docx]

**Supplementary Table S10. Top 20 REACTOME gene sets from GSEA of down-regulated and up-regulated genes in PG1**

| Gene set | Overlap | Size | pval | FDR |
| --- | --- | --- | --- | --- |
| **Top 20 REACTOME gene sets from GSEA of down-regulated genes in PG1** |  |  |  |  |
| REACTOME_BIOLOGICAL_OXIDATIONS | 53 | 219 | 6.58E-40 | 1.06E-36 |
| REACTOME_PHASE_I_FUNCTIONALIZATION_OF_COMPOUNDS | 32 | 104 | 3.20E-28 | 2.56E-25 |
| REACTOME_METABOLISM_OF_AMINO_ACIDS_AND_DERIVATIVES | 42 | 375 | 2.91E-18 | 1.56E-15 |
| REACTOME_METABOLISM_OF_LIPIDS | 59 | 742 | 8.02E-18 | 3.21E-15 |
| REACTOME_CYTOCHROME_P450_ARRANGED_BY_SUBSTRATE_TYPE | 19 | 66 | 1.06E-16 | 3.39E-14 |
| REACTOME_FATTY_ACID_METABOLISM | 26 | 178 | 1.48E-14 | 3.97E-12 |
| REACTOME_PHASE_II_CONJUGATION_OF_COMPOUNDS | 19 | 108 | 1.74E-12 | 3.48E-10 |
| REACTOME_SYNTHESIS_OF_BILE_ACIDS_AND_BILE_SALTS_VIA_24_HYDROXYCHOLESTEROL | 9 | 14 | 1.65E-12 | 3.48E-10 |
| REACTOME_BILE_ACID_AND_BILE_SALT_METABOLISM | 13 | 43 | 3.67E-12 | 6.54E-10 |
| REACTOME_SYNTHESIS_OF_BILE_ACIDS_AND_BILE_SALTS_VIA_7ALPHA_HYDROXYCHOLESTEROL | 10 | 24 | 2.87E-11 | 4.19E-09 |
| REACTOME_ARACHIDONIC_ACID_METABOLISM | 14 | 60 | 2.67E-11 | 4.19E-09 |
| REACTOME_COMPLEMENT_CASCADE | 18 | 114 | 4.39E-11 | 5.79E-09 |
| REACTOME_XENOBIOTICS | 10 | 25 | 4.69E-11 | 5.79E-09 |
| REACTOME_SYNTHESIS_OF_BILE_ACIDS_AND_BILE_SALTS | 11 | 34 | 7.42E-11 | 8.50E-09 |
| REACTOME_FATTY_ACIDS | 8 | 15 | 2.41E-10 | 2.57E-08 |
| REACTOME_METABOLISM_OF_STEROIDS | 19 | 151 | 7.06E-10 | 7.07E-08 |
| REACTOME_AMINO_ACID_CONJUGATION | 6 | 9 | 7.52E-09 | 6.70E-07 |
| REACTOME_SYNTHESIS_OF_16_20_HYDROXYEICOSATETRAENOIC_ACIDS_HETE | 6 | 9 | 7.52E-09 | 6.70E-07 |
| REACTOME_TRANSPORT_OF_SMALL_MOLECULES | 42 | 729 | 9.88E-09 | 8.34E-07 |
| REACTOME_CYP2E1_REACTIONS | 6 | 11 | 3.99E-08 | 3.20E-06 |
| **Top 20 REACTOME gene sets from GSEA of up-regulated genes in PG1** |  |  |  |  |
| REACTOME_NEURONAL_SYSTEM | 15 | 410 | 9.08E-06 | 0.004998 |
| REACTOME_SIGNALING_BY_GPCR | 21 | 699 | 3.49E-06 | 0.004998 |
| REACTOME_GPCR_LIGAND_BINDING | 16 | 463 | 9.35E-06 | 0.004998 |
| REACTOME_POTASSIUM_CHANNELS | 7 | 103 | 5.43E-05 | 0.017418 |
| REACTOME_G_ALPHA_I_SIGNALLING_EVENTS | 12 | 314 | 4.67E-05 | 0.017418 |
| REACTOME_EXTRACELLULAR_MATRIX_ORGANIZATION | 11 | 301 | 0.000142 | 0.038037 |
| REACTOME_ACTIVATION_OF_KAINATE_RECEPTORS_UPON_GLUTAMATE_BINDING | 4 | 30 | 0.00017 | 0.039003 |
| REACTOME_CHEMOKINE_RECEPTORS_BIND_CHEMOKINES | 5 | 57 | 0.000196 | 0.039234 |
| REACTOME_CLASS_B_2_SECRETIN_FAMILY_RECEPTORS | 6 | 94 | 0.000262 | 0.04669 |
| REACTOME_INTEGRATION_OF_ENERGY_METABOLISM | 6 | 108 | 0.000552 | 0.088537 |
| REACTOME_RND1_GTPASE_CYCLE | 4 | 42 | 0.000636 | 0.092804 |
| REACTOME_VOLTAGE_GATED_POTASSIUM_CHANNELS | 4 | 43 | 0.000697 | 0.093109 |
| REACTOME_REGULATION_OF_INSULIN_SECRETION | 5 | 78 | 0.000841 | 0.103735 |
| REACTOME_TRANSMISSION_ACROSS_CHEMICAL_SYNAPSES | 9 | 269 | 0.001054 | 0.106136 |
| REACTOME_PRESYNAPTIC_FUNCTION_OF_KAINATE_RECEPTORS | 3 | 21 | 0.000952 | 0.106136 |
| REACTOME_METABOLISM_OF_FAT_SOLUBLE_VITAMINS | 4 | 48 | 0.001059 | 0.106136 |
| REACTOME_CLASS_A_1_RHODOPSIN_LIKE_RECEPTORS | 10 | 331 | 0.001225 | 0.115541 |
| REACTOME_ANTI_INFLAMMATORY_RESPONSE_FAVOURING_LEISHMANIA_PARASITE_INFECTION | 8 | 224 | 0.001311 | 0.116844 |
| REACTOME_SYNTHESIS_OF_VERY_LONG_CHAIN_FATTY_ACYL_COAS | 3 | 24 | 0.001419 | 0.119827 |
| REACTOME_ADORA2B_MEDIATED_ANTI_INFLAMMATORY_CYTOKINES_PRODUCTION | 6 | 133 | 0.001628 | 0.130597 |
