# Supplementary material for: Multi-region sampling with paired sample sequencing analyses reveals sub-groups of patients with novel patient-specific dysregulation in Hepatocellular Carcinoma
Source: BMC Cancer. 2023 Feb 3;23:118. doi: 10.1186/s12885-022-10444-3 (PMC9896715; doi:10.1186/s12885-022-10444-3)
Supplement: Supplementary file 9 — Additional file 9: Fig. S1. Number of up-regulated (top) and down-regulated (bottom) genes per each DEPC value, using randomly assigned adj.normal samples. Coloured lines represent mean±s.d. after 100 iterations. Black line represents the values with the original N-T pairing for each patient. [file 12885_2022_10444_MOESM9_ESM.pdf]

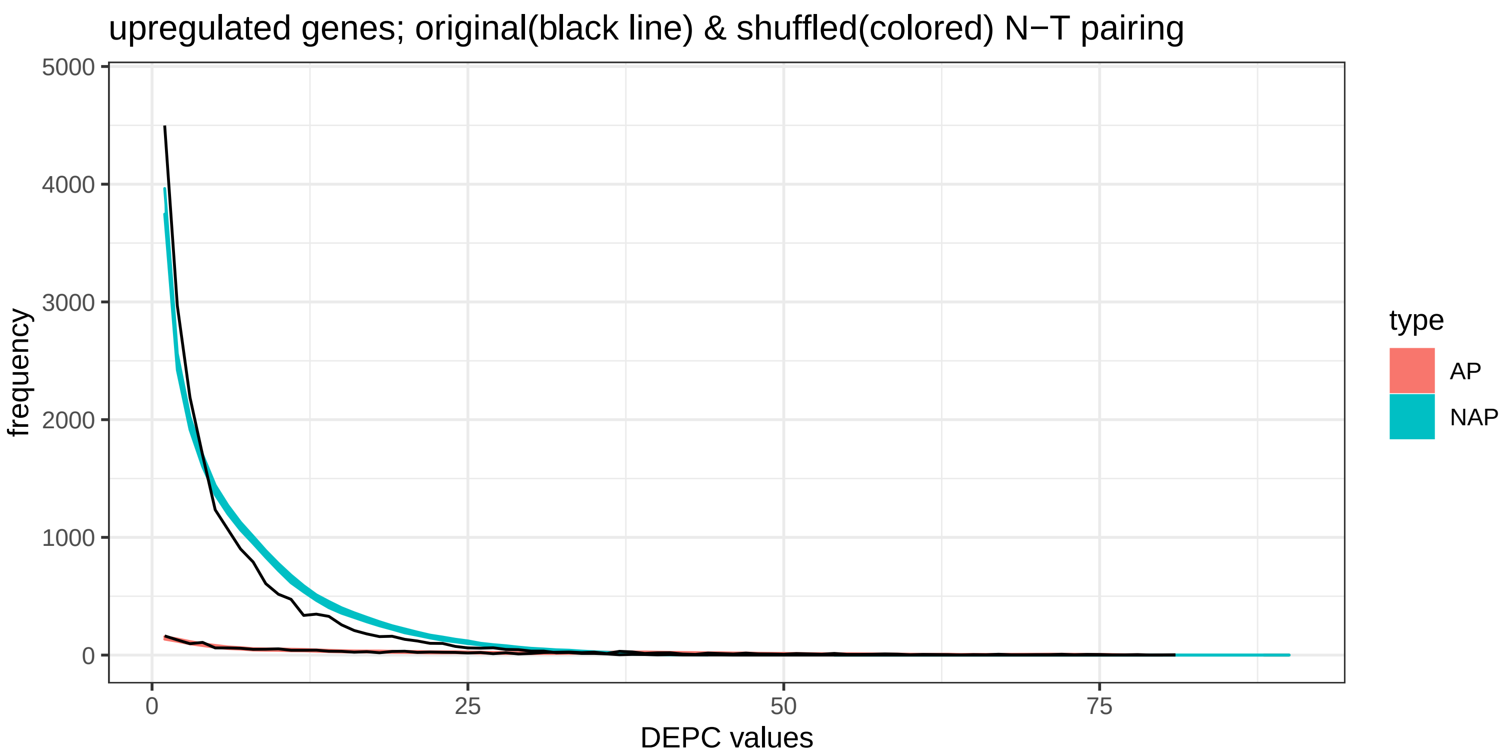
upregulated genes; original(black line) & shuffled(colored) N−T pairing
5000
4000
3000
2000
1000
0
frequency
0
25
50
75
DEPC values
type
AP
NAP

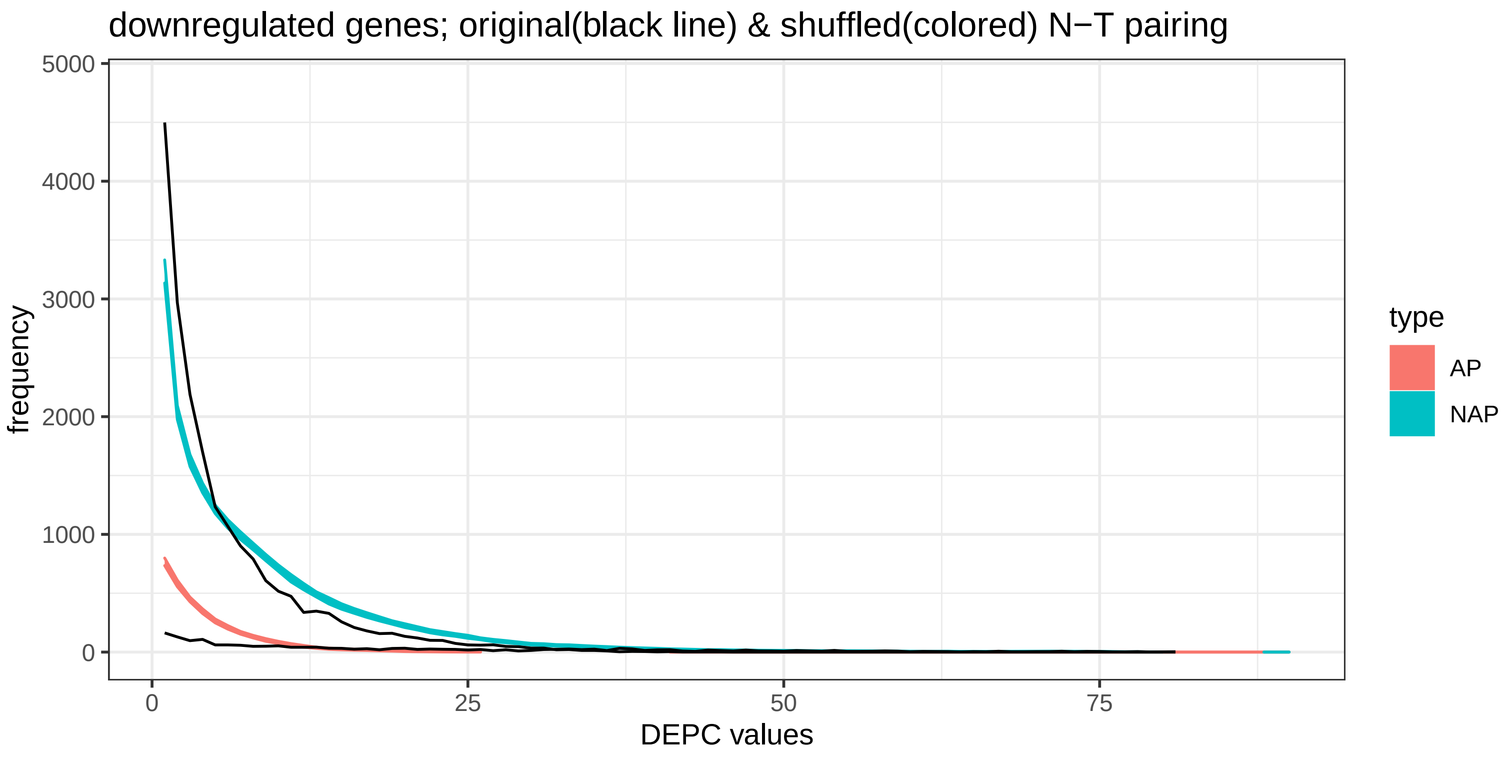
downregulated genes; original(black line) & shuffled(colored) N−T pairing
5000
4000
3000
2000
1000
0
frequency
0
25
50
75
DEPC values
type
AP
NAP
