# Supplementary figures and images for: Multi-region sampling with paired sample sequencing analyses reveals sub-groups of patients with novel patient-specific dysregulation in Hepatocellular Carcinoma
Source: BMC Cancer. 2023 Feb 3;23:118. doi: 10.1186/s12885-022-10444-3 (PMC9896715; doi:10.1186/s12885-022-10444-3)

**A**

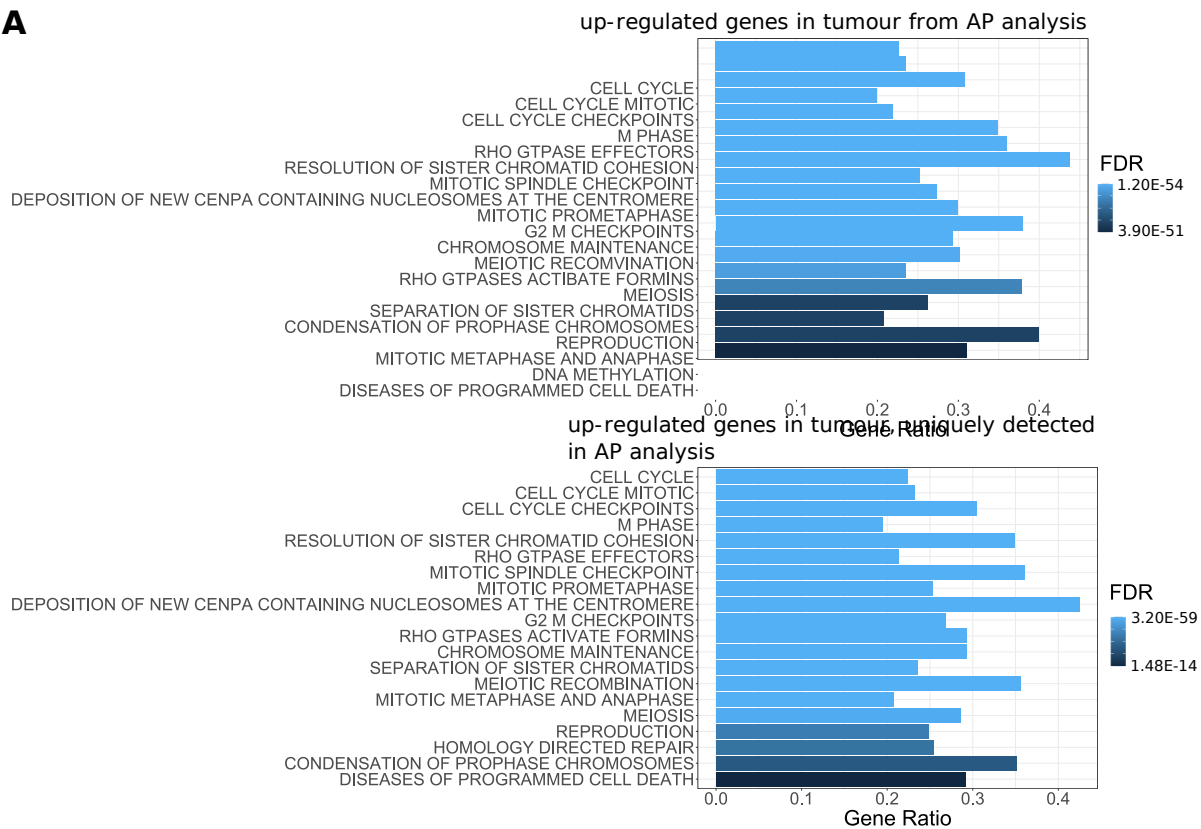

**B**

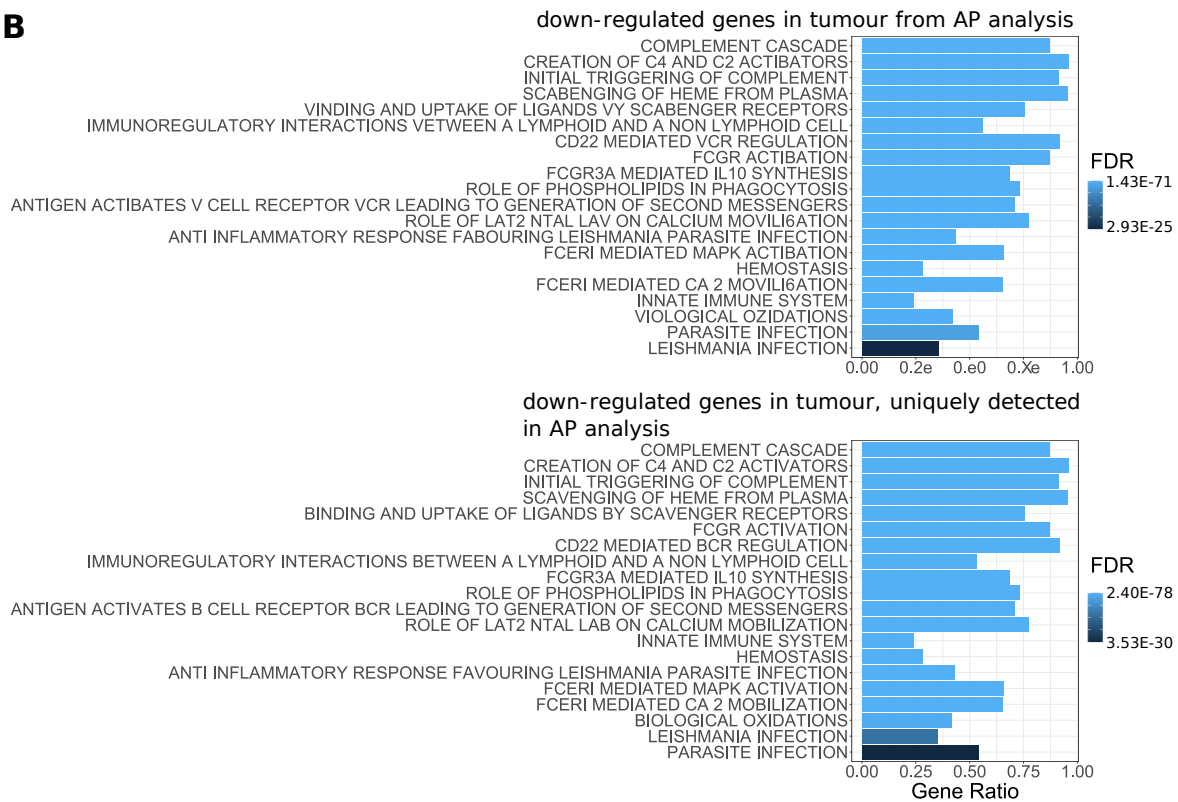

Supplement: Supplementary file 10 — Additional file 10: Fig. S2. A. GSEA results for up-regulated genes in tumour tissues from all-patients (AP) analysis. B: GSEA results for down-regulated genes in tumour tissues from AP analysis. Across both panels, top chart shows results including all the up-regulated genes from the AP analysis, while the bottom chart excludes the genes also detected in PP analyses. [file 12885_2022_10444_MOESM10_ESM.pdf]

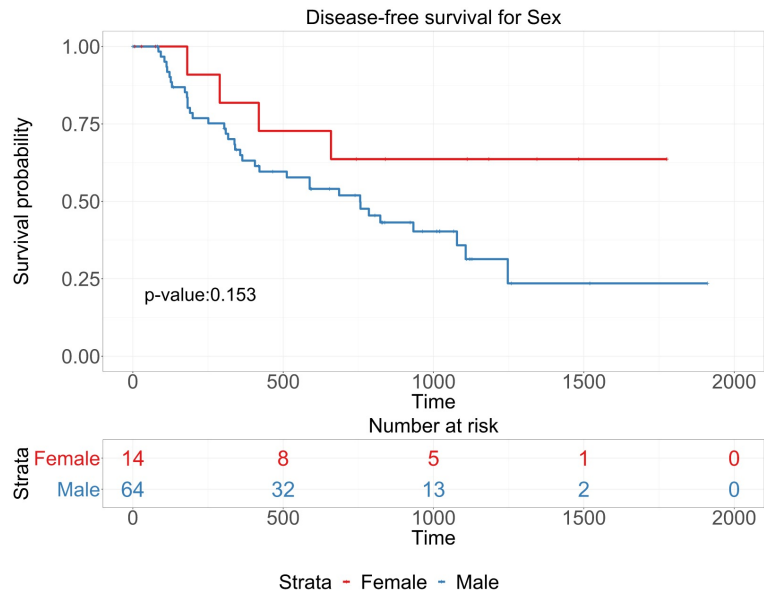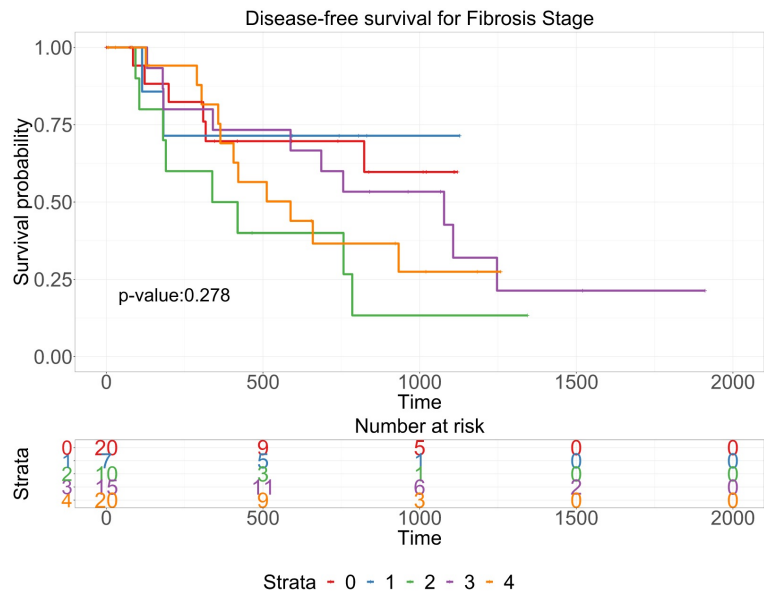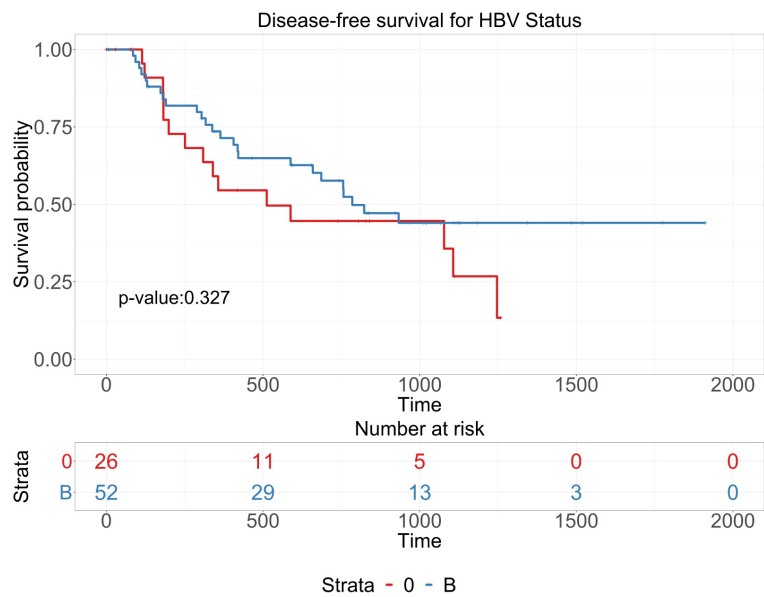

Supplement: Supplementary file 11 — Additional file 11: Fig. S3. Kaplan-Meier recurrence-free survival plots for patients that are categorized based on sex(top), fibrosis stages (middle), and HBV status (bottom). [file 12885_2022_10444_MOESM11_ESM.pdf]

A

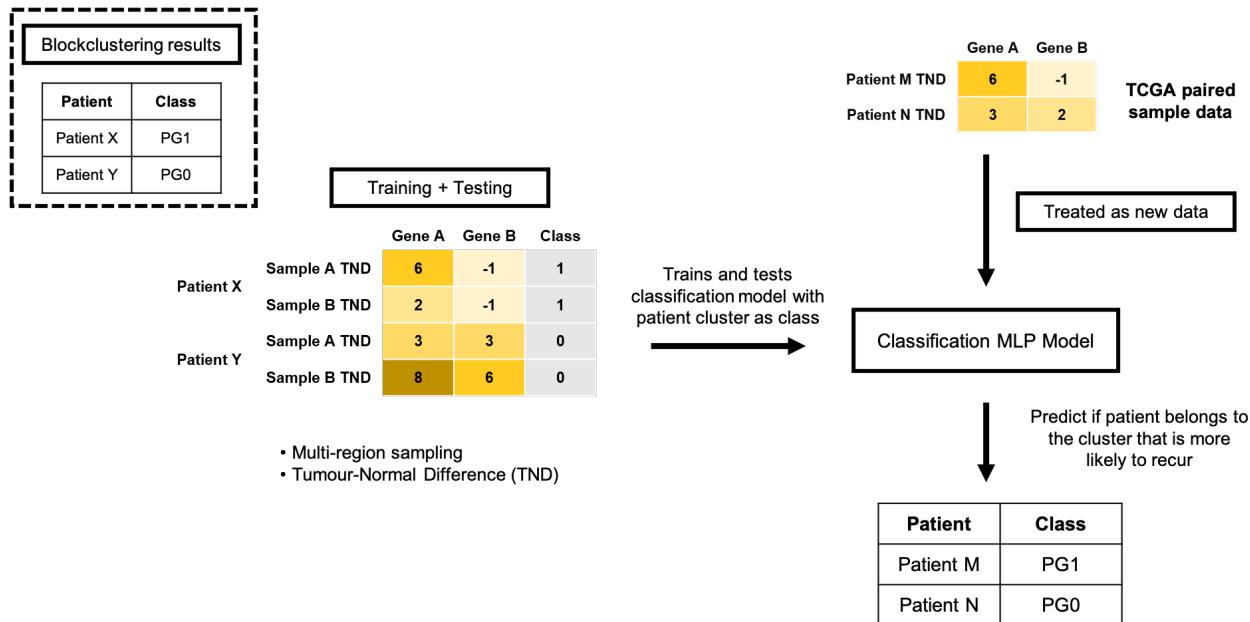

B

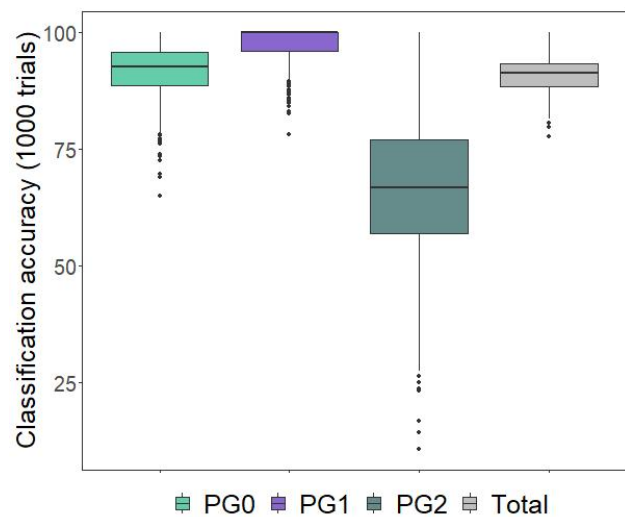

Supplement: Supplementary file 12 — Additional file 12: Fig. S4. A. Schematic illustration of machine-learning based patient stratification strategy for TCGA- LIHC paired sample data. B: Patient stratification classification accuracies of PG0, PG1, PG2 and combined (Total). [file 12885_2022_10444_MOESM12_ESM.pdf]
